# The Efficacy of the RME II System Compared with a Herbst Appliance in the Treatment of Class II Skeletal Malocclusion in Growing Patients: A Retrospective Study

**DOI:** 10.3390/dj12080254

**Published:** 2024-08-13

**Authors:** Domenico Ciavarella, Mauro Lorusso, Carlotta Fanelli, Donatella Ferrara, Rosa Esposito, Michele Laurenziello, Fariba Esperouz, Lucio Lo Russo, Michele Tepedino

**Affiliations:** 1 Department of Clinical and Experimental Medicine, Dental School of Foggia, University of Foggia, 71122 Foggia, Italy; domenico.ciavarella@unifg.it (D.C.); carlotta.fanelli@unifg.it (C.F.); donatella.ferrara@unifg.it (D.F.); michele.laurenziello@unifg.it (M.L.); fariba.esperouz@unifg.it (F.E.); lucio.lorusso@unifg.it (L.L.R.); 2 Department of Biotechnological and Applied Clinical Sciences, Dental School of L’Aquila, University of L’Aquila, 67100 L’Aquila, Italy; rosa.esposito1@graduate.univaq.it (R.E.); michele.tepedino@univaq.it (M.T.)

**Keywords:** RME II System, Herbst appliance, class II malocclusion, growing patients

## Abstract

(1) Background: The objective of this study was to evaluate the efficacy of the Rapid Maxillary Expander (RME) II System compared to a Herbst appliance and a control group in the treatment of class II skeletal malocclusions in growing patients. (2) Methods: A total of 30 class II patients treated using the RME II System (group R) were compared with 30 patients treated with a Herbst appliance (group H) and 30 untreated class II children (group C). Cephalograms were compared at the start (T0) and after 24 months (T1). Nine cephalometric parameters were analyzed: SN-MP, SN-PO, ANB, AR-GO-ME, AR-GO-N, N-GO-ME, SN-PP, LFH, CO-GN, 1+SN, IMPA, OVERJET, and OVERBITE. Since the variables failed the normality test, a Wilcoxon test was performed for a pairwise comparison of the cephalometric measurements taken at T0 (pre-treatment) and at T1 (post-treatment). ANOVA with Tukey post hoc correction was used to evaluate the differences among the groups. (3) Results: ANOVA showed a statistically significant difference for all analyzed variables except for AR-GO-ME, AR-GO-N, and N-GO-ME. Post hoc Tukey’s HSD test showed the following difference: the SN-PO angle in group H was 3.59° greater than in group R; the LFH in group H was 4.13 mm greater than in group R. The mandibular length (CO-GN) in group H was 3.94 mm greater than in group R; IMPA in group H was 6.4° greater than in group R; and the ANB angle in group H was 1.47° greater than in group R. (4) Conclusions: The RME II System is an effective therapeutic device for class II skeletal malocclusion treatment in growing patients.

## 1. Introduction

Class II malocclusion is a jaw discrepancy in which multiple factors contribute to its etiology, including genetic, skeletal, and dental components. In 1899, Angle defined normal occlusion as the relationship between the first upper and lower molars so that the mesio-buccal cusp of the upper molar occludes in the buccal groove of the lower molar [1]. The World Oral Health considers malocclusions as the third greatest oral health problem after caries and periodontal disease [2]. Class II skeletal malocclusion in growing patients is commonly treated with functional orthodontic appliances that guide the jaws into a better position, changing muscle conditions and reducing the discrepancy [3,4]. Functional treatment is more predictable during the pubertal growth peak with more skeletal effects than in the pre- or post-pubertal period [5,6]. Malocclusions involve the three spatial planes: sagittal, transverse, and vertical. Therefore, in the treatment of class II malocclusions, in addition to sagittal discrepancy, alterations in the vertical and transverse planes must also be considered.

The early treatment of class II malocclusion can improve patients’ quality of life since severe mandibular retrognathism is linked to obstructive sleep apnea (OSA) due to a posterior displacement of the tongue that leads to airway constriction [7,8]. Therapeutic mandibular advancement with functional appliances improves OSA parameters, so it is reasonable that functional appliance therapy might be associated with beneficial effects on the upper airway [9]. Therefore, early treatment with functional appliances could decrease the potential risk of OSA in growing patients through increased space in the oropharyngeal airway [10].

Class II malocclusion can modify the perioral muscle pattern. Frequently, altered activity of mentalis and buccinator muscles associated with the tongue and lip compensatory position is observed. These alterations may cause maxillary constriction, altered incisor inclinations, and soft tissue abnormalities with a typical convex profile [11]. In class II patients, an altered position of the condyle and disk is observed [12], although studies are discordant [13]. Moreover, a posterior dislocation of the condyle and an anterior dislocation of the meniscus associated with joint click has been found in class II skeletal patients [14]. For these reasons, in growing patients with class II malocclusion, early treatment is required in order to correct skeletal discrepancies.

The Herbst appliance is one of the most widely used fixed functional devices in class II malocclusion therapy. Placed between the maxillary and mandibular dental arches, it utilizes a bilateral telescopic mechanism that keeps the mandible in an advanced position. The Herbst appliance used in the present study had the telescopic tube attached to the band of the maxillary first permanent molar and the telescope plunger attached to the mandibular canine. Anchorage in the upper dental arch consisted of a palatal or buccal sectional arch wire connecting the first molar to the first premolar.

The “RME II System” is a functional device for the treatment of class II malocclusion with concomitant maxillary transverse discrepancy. The appliance consists of a Hyrax maxillary expander anchored on the deciduous second molars, with two arms extending to the canines and a lower lingual arch to which class II elastics are fitted. This orthodontic appliance used in the Orthodontic Department of the research institution allows for the correction of sagittal and transverse discrepancies in patients with class II skeletal malocclusion. The purpose of the device design is to reduce discomfort and undesirable effects associated with the use of other orthodontic fixed appliances, such as the Herbst appliance [15], by achieving orthopedic effects in growing patients.

The aim of this study was to evaluate the efficacy of the RME II System in class II correction, compared with the Herbst appliance in the treatment of class II skeletal malocclusions in growing patients. Therefore, the main outcome of this study was to analyze and compare the effects of the devices used in the different groups in order to understand the various skeletal and dental mechanisms of action resulting from the treatment. The secondary outcome of this study was assessing the impact of the therapeutic treatment of each device used by analyzing the skeletal and dental differences before and after treatment.

## 2. Material and Methods

This study was conducted in accordance with the Strengthening the Reporting of Observational Studies in Epidemiology (STROBE) guidelines [16].

The procedures in this research protocol adhered to the Declaration of Helsinki and received approval from the Institutional Ethics Committee. Medical records were retrieved retrospectively and analyzed in an anonymous form. Written informed consent was obtained from the patients’ parents. The inclusion and exclusion criteria are described in Table 1.

According to a power analysis conducted using G*Power 3.1.9.2 (Franz Faul, Universität Kiel, Germany), 28 subjects per group are required to detect a large effect size of 0.4 [17] with a one-way ANOVA test, an α significance level of 0.05, and a power (1 − β error probability) of 0.90.

The sample consisted of three groups: one group composed of patients treated with the RME II System (group R), one group composed of patients treated with a Herbst appliance (group H), and one group composed of untreated controls (group C). Groups R and H were retrospectively formed from patients treated at the Department of Orthodontics, University of Foggia, Italy, between March 2017 and October 2019, in chronological order. The treatment was concluded once a class I molar and canine relationship was achieved. Group C was sampled from the Michigan Library patients. The three samples exhibited similar dentoskeletal characteristics at baseline (T0), although the control group had more upright lower incisors. Pre-treatment (T0) and post-treatment (T1) records comprised study models, photographs, panoramic radiographs, and lateral cephalograms.

### 2.1. Group R

Group R consisted of 30 patients (12 males and 18 females, mean age of 9.4, and SD of 0.6 years). The mean treatment time was 12 months (range 9–15).

**Figure 1:**
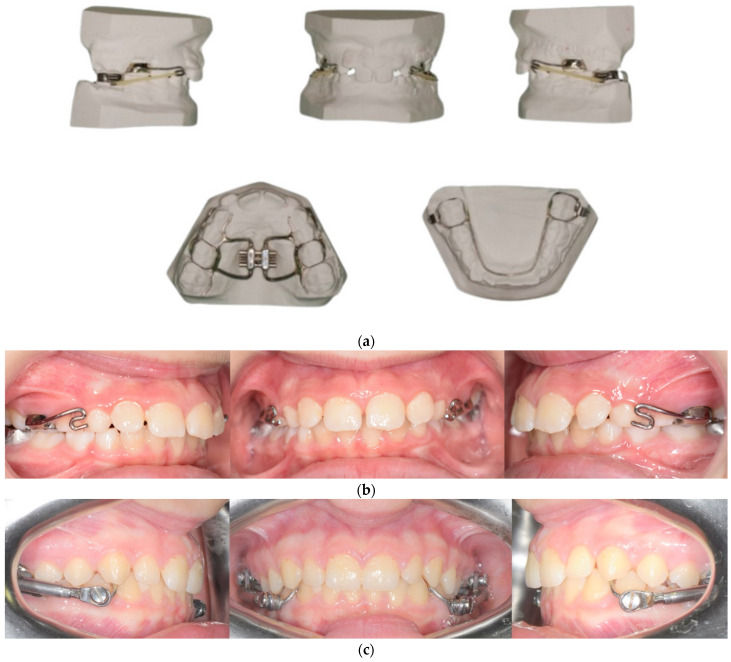
(**a**). The RME II System on a plaster model with intermaxillary elastics; (**b**) the clinical photos of the RME II System; (**c**) the clinical photos of the Herbst appliance.

The RME II System is a functional device consisting of a Hyrax-type expander anchored on the deciduous second molars, with two rigid vestibular arms extended to the canines and a lower lingual arch with hooks to which intermaxillary elastics are anchored. Figure 1a shows the Rep II system on a plaster model with intermaxillary elastics, and Figure 1b shows the clinical photos of the RME II System. The expander was activated once every 21 days (very slow maxillary expansion), and patients were trained to use class II elastics for 16 h a day. The protocol for elastic usage was as follows: 4.5 oz and 3/8” for 4 months, then 6 oz and 3/8” for one month and finally 4 oz and 3/8” for 2 months. The elastics were changed every day to provide maximum traction efficacy.

During treatment, the loss of deciduous molars occurred early in two cases that were not included in this study. In five cases, the loss occurred at the end of treatment, and it did not affect therapy. In eleven cases, an increase in mobility was observed during the eighth month of therapy. However, treatment could continue because the class correction had already been achieved, the transverse discrepancy had been corrected, and exfoliation occurred three months after the end of treatment.

### 2.2. Group H

Group H comprised 30 patients (15 males and 15 females, mean age or 9.2, and SD of 0.7 years) who were treated using the Herbst appliance. The mean treatment time was 12 months (range 8–14).

Figure 1c shows the clinical photos of the Herbst appliance.

### 2.3. Group C

Group C consisted of 30 patients (16 males and 14 females, mean age of 9.9, and SD of 0.4 years). These patients received no treatment. These patients were recruited from the Michigan Medical Library and selected by age and gender in order to be comparable with the other two groups analyzed.

### 2.4. Cephalometric Analysis

Lateral cephalograms (Gendex GXDP-700, GENDEX DENTAL SYSTEMS S.R.L., Varese, Italy) were obtained with the head of the patient positioned in a cephalostat, in centric occlusion, ensuring the clear visualization of the landmark structures and no head rotation.

**Figure 2:**
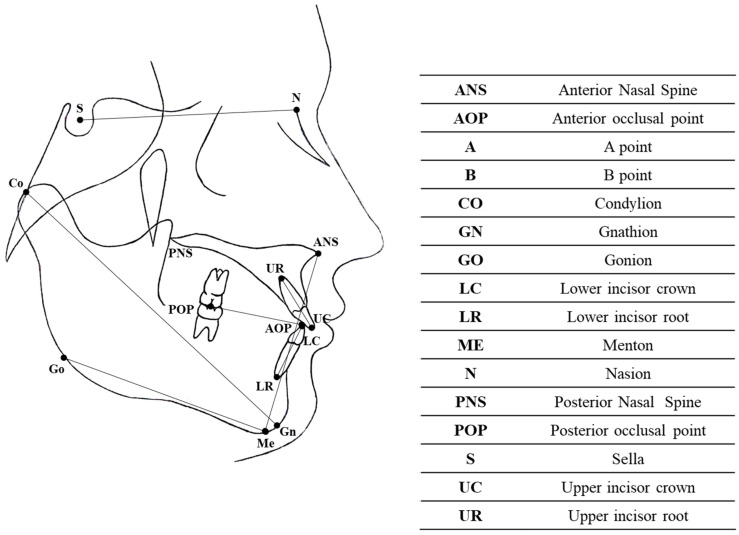
Cephalometric landmarks and reference lines.

Lateral radiographs were made by the same technician and with the same machine in the same radiology department. Cephalometric analysis was conducted on the lateral cephalograms. The following cephalometric skeletal and dental variables were analyzed: SN-MP, ANB, SN-PO, AR-GO-ME, AR-GO-N, N-GO-ME, SN-PP, LFH (lower face height), CO-GN, 1+SN, IMPA, OVERJET, and OVERBITE. The landmarks and reference lines employed for the cephalometric analysis are shown in Figure 2 and detailed in Table 2. To reduce the error of the method, cephalometric analyses were conducted by an experienced orthodontist, and all measurements were taken twice by the same provider.

### 2.5. Statistical Analysis

To minimize random errors, cephalometric and dental measurements were performed two times. The random error of measurements was determined using Dahlberg’s formula (*S* = ∑ *d*^2^/2*N*), where *d* represents the difference between the first and second measurements, and *N* is the number of radiographs assessed [18,19]. The random error was 0.32 to 0.51 mm for linear measurements and 0.38° to 0.56° for angular measurements.

The Shapiro–Wilk normality test was conducted to assess the trend in the data. Because the variables failed the normality test, a Wilcoxon signed-rank test (Table 3) was used for the pairwise comparison of the cephalometric measurements taken at T0 (pre-treatment) and at T1 (post-treatment) within each group. The data were examined using GraphPad Prism software 6.0 (GraphPad Prism Software, San Diego, CA, USA). The differences among the groups were first evaluated through a one-way ANOVA test for the T1 − T0 difference in each respective variable and finally with Tukey’s post hoc test (Table 4 and Table 5). Statistical significance was set as *p* < 0.05.

## 3. Results

The findings of the present study can be summarized as follows:

- Patients treated with the RME II System showed a decreased lower incisor inclination, decreased occlusal plane inclination, a greater reduction in the ANB angle and LFH, and decreased mandibular length compared with patients treated with the Herbst appliance;
- Patients treated with the RME II System showed an increased mandibular length, reduced overjet and overbite, a smaller divergence angle and LFH, and a greater occlusal plane inclination compared with the control group;
- Patients treated with the Herbst appliance showed a greater occlusal plane inclination and SN-PP angle, increased lower incisor proclination, and a reduction in overjet, overbite, and upper incisor inclination compared with the control group.

## 4. Discussion

This study aimed to compare the dentoskeletal effects of the RME II System and the Herbst appliance in class II malocclusion patients compared to an untreated control group. A Herbst appliance modified by Pancherz [20] has been used to correct sagittal discrepancies due to the mandibular retroposition. This appliance is very efficient [21,22,23] in correcting the mandibular position thanks to the bite jump efficacy in stimulating jaw growth and rebalancing soft tissue. Bock et al. [24] in a meta-analysis observed that, in patients treated with a Herbst appliance, dentoskeletal effects were stable without significant clinical changes over time. However, different limitations were evident when using the Herbst appliance; one of the main problems was related to the headgear effect, resulting in upper molar distalization and intrusion and a consequent increase in facial divergence due to mandibular molar extrusion [15,25]. Nevertheless, the effects of the Herbst appliance on mandibular growth and soft tissue changes are still unclear [26].

In the present study, we observed a significant increase in the LFH and SN-PO angle in group H compared to group R, in agreement with Pancherz et al. [27]. This difference was also observed for the SN-PO angle between the Herbst and control groups. These effects were probably due to lower molar extrusion resulting from upper molar intrusion and the consequent occlusal plane rotation. The same mechanism was probably behind the observed decrease in overbite in group H compared to group C, in line with the previous observation by Pancherz et al. [27]; another important difference was observed for the significantly increased IMPA in group H compared to groups R and C. This effect was due to the loss of anterior anchorage in the lower arch, resulting in incisor proclination and consequent overjet reduction, as also observed by Barnett et al. [25]. To overcome this problem, some authors have suggested the use of a rigid lower splint anchored on mini-screws [28,29]. However, this modification reduces the loss of anchorage but does not eliminate it completely, as observed by Manni et al. [30]; moreover, patients’ parents often reject the use of mini-screws. Regarding the mandibular length measured through the CO-GN distance, group H showed a significant increase with respect to group R. Different studies have reported an increase in mandibular length after Herbst treatment [22,31], in line with the results of the present study. In addition, in the present study, an increase in mandibular length was observed in patients in group R compared to the control group.

Regarding the divergence angle (SN-MP), statistical analysis showed a reduction in group R compared to group C; this effect was probably due to the anchorage on deciduous upper molars that did not cause tipping, and the lower lingual arch which controls the mandibular molar extrusive effect.

Regarding group H, statistical analysis showed a reduction in upper incisor inclination (1+SN) compared to group C; this is in contrast with the results obtained by Flores-Mir et al. [32] who did not observe changes in upper incisor inclination after Herbst therapy. In addition, an increased SN-PP angle in group H was observed compared to group C. For the ANB angle, the results showed a decrease in group R compared to group C and an increase in group H compared to group R. Several studies have shown mandibular forward displacement after Herbst therapy [25,27] and although these changes were stable in the long term [24], patients treated with the RME II System seemed to have a greater mandibular displacement benefit. Therefore, it is possible to postulate that the control over the position of the lower incisor, occlusal plane control, and increase in the transversal arch diameter offered by the RME II System resulted in greater mandibular anterior repositioning. It is very important to control the occlusal plane because, when it rotates in the clockwise direction, the B point is displaced backward, contributing to the worsening of malocclusion.

The expander was cemented onto the deciduous molars because the timing of exfoliation, assessed by OPT, allowed us to avoid support on the permanent molars. This resulted in pure orthopedic effects without dental compensation, thus reducing the side effects associated with expansion on permanent teeth (root resorption, bone loss, and gingival recession) [33,34]. Class II elastics used in fixed orthodontics can exhibit dental compensation effects because they are applied to permanent teeth. In the present study, dental compensation was not observed because both upper and lower anchorage were used. The elastics had negligible dental effects because they were applied to the superior expander hook anchored on the deciduous molars and to the lingual arch hook in the mandible. Moreover, as seen in Figure 1b, the upper hook was positioned very low, almost at the level of the cusp of the deciduous canine. This positioning was designed to minimize the vertical force vector, thereby favoring the action of the vector in the sagittal plane. Finally, the lingual arch significantly reduces the undesirable effects of class II elastics, especially on the lower incisors, thereby promoting better repositioning of the mandible.

The results of the present study revealed the main limitation of the Herbst appliance: the loss of anchorage resulting in the proclination of the lower incisors, which reduces overjet and consequently mandibular advancement. In fact, Herbst’s appliance class correction is related to the combined action of upper molar distalization and mandibular advancement. Although it is possible to manage these limitations with miniscrews, it should be noted that it is not always possible to use them, and in such cases, the limitations of this device must be taken into account. Finally, it should be considered that palatal expansion could be a confounder in the correction of class II, so it may have partly influenced the results of this study. Consequently, in the future, both devices should be compared after the palatal expansion phase.

The differences shown in this study can also be explained by the distinct designs of the two devices. The Herbst appliance features telescopic arms that rest on the mandibular canines, resulting in a direct impact on the proclination of the lower incisors. Additionally, the force vector induces a distalizing and intrusive effect at the level of the upper molars, explaining the observed changes in occlusal plane and lower facial height. The efficacy of the RME II System can be attributed to both the expander, which facilitates the unblocking of mandibular dystoposition, and the action of the class elastics, which is used with a lingual arch to control the extrusion of the lower molars.

The differences observed in the present study are clinically significant. The control of the inclination of the lower incisors and the occlusal plane is of crucial importance in the therapy for class II malocclusion since the reduction in the overjet is associated with reduced mandibular advancement; in addition, the rotation of the occlusal plane can lead to complications such as the occurrence of an open bite, which, in the case of hyperdivergent subjects, can be very complicated to manage. For these reasons, the RME II System makes it possible to restore the correct sagittal skeletal relationships by reducing the undesirable effects typical of functional devices used in class II malocclusion therapy.

The ideal timing for the treatment of class II malocclusions, according to the literature, is during peak pubertal growth [35]. However, the guidelines define the need for early treatment in cases of increased overjet and upper incisor inclination, which are considered risk factors in the case of trauma [36]. For these reasons, it was necessary to intervene early and treat these patients before reaching peak pubertal growth.

As observed by Baratieri et al. [37], the effects of maxillary expansion can facilitate the repositioning of the mandible by improving the sagittal relationships between the maxilla and mandible. The RME II System takes advantage of this effect and, in combination with class II elastics, promotes the correction of malocclusion.

### Limitations of the Study

The limitations of this study are the retrospective nature of patient recruitment and the use of a historical untreated control group. As group C was recruited from the Michigan Medical Library, the ethnic, socio-cultural, and genetic characteristics may differ from those of patients in the other two groups. A further limitation is related to the lack of follow-up to confirm the treatment’s stability and to the bi-dimensional characteristics of the cephalometric exam used. Due to the retrospective nature of this study, it is difficult to understand which other unanalyzed variables might have influenced the relationship between the cephalometric measurements among the groups. An important limitation is related to the lack of condyle position assessment in treated patients in order to understand the relationship between the mandibular position and sagittal correction.

The time factor is an important aspect, as the effectiveness of the RME II System is dependent on the timing of dentition. In the case of permanent teeth, the effects could result in dental compensation.

Finally, the selection of the ANB angle to assess mandibular advancement may have limitations since this angle is influenced by cranial base orientation. Future studies should implement a long-term follow-up.

## 5. Conclusions

According to the results of the present study, the RME II System controls vertical effects better compared to the Herbst appliance, so this device can represent a therapeutic alternative for the treatment of class II malocclusion. The advantages of this appliance may be related to expansion effects that lead the mandible to a better position, improving class II relationship. Another important effect is related to the use of elastics on the lingual arch which reduces the undesirable extrusive effects on molars and consequent occlusal plane rotation. The limitation of this appliance, compared to the Herbst, is related to patient compliance in class II elastics use.

## Figures and Tables

**Table 1:** Inclusion and exclusion criteria.

Inclusion Criteria	Exclusion Criteria
Complete eruption of permanent incisors and first permanent molars	Mono- or bilateral crossbite
Age between 9 and 13 years	Patients with complete permanent teeth
Class II division 1 malocclusion	Early loss of second deciduous molars
Lateral cephalogram performed with the same cephalostat	Skeletal malformations and destructive caries
Skeletal age between CS2 and CS3 according to the cervical vertebral maturation method	Previous cervical trauma
Absence of temporomandibular joint disorders	Patients with previous orthodontic treatment
No maxillofacial or airway surgery	

**Table 2:** Cephalometric measurements.

Measurement	Description
Skeletal measurements	
SN-MP	Angle between the sella–nasion (SN) line and the mandibular plane (MP)
SN-PO	Angle between the sella–nasion (SN) line and the occlusal plane (APO–PPO)
ANB	Angle between the N–A line and N–B line
AR-GO-ME	Gonial angle: the angle between the AR point and the mandibular plane (MP)
UPPER GONIAL ANGLE (AR-GO-N)	Angle between the N–AR line and the AR–GO line
LOWER GONIAL ANGLE (N-GO-ME)	Angle between the gonion–nasion (GO–N) line and the gonion–menton (GO–ME) line
SN-PP	Angle between the sella–nasion (SN) line and the maxillary plane (ANS–PNS)
LFH (lower face height)	Distance between the anterior nasal spine (ANS) and the menton (ME)
MANDIBULAR LENGTH (CO-GN)	Distance between the condylion and the gnathion
Dental measurements	
1+SN	Angle between the upper incisor and the sella–nasion line
IMPA	Angle between the lower incisor and the mandibular plane (GO–ME)
OVERJET	Horizontal distance between the incisal border of the upper and lower incisors
OVERBITE	Vertical distance between the incisal border of the upper and lower incisors

**Table 3:** Wilcoxon signed-rank test between cephalometric variables taken at T0 and at T1 within the three groups (in bold are the statistically significant correlations).

	Group R					Group H					Group C				
	T0		T1		P	T0		T1		P	T0		T1		P
	Mean	Std Dev.	Mean	Std Dev.		Mean	Std Dev.	Mean	Std Dev.		Mean	Std Dev.	Mean	Std Dev.	
SN-PO	19.90	4.21	19.26	4.63	*0.18*	14.36	4.43	17.32	8.01	*0.264*	15.98	3.24	12.07	4.02	***0.001***
SN-MP	35.21	4.70	32.67	4.81	***0.001***	34.03	3.30	32.81	5.79	***0.041***	34.61	3.76	33.76	3.95	***0.05***
AR-GO-ME	124.5	6.41	124.3	7.43	*0.264*	132.9	7.91	131.5	7.04	*0.431*	134.7	6.06	132.6	5.55	*0.614*
AR-GO-N	51.62	3.40	51.75	4.70	*0.362*	58.41	3.91	56.82	5.23	*0.752*	58.88	2.83	57.89	3.96	*0.158*
N-GO-ME	72.85	4.84	72.36	4.48	*0.451*	74.9	4.95	74.62	4.50	*0.461*	75.81	4.60	74.75	3.85	*0.219*
SN-PP	9.08	4.16	9.43	4.94	*0.753*	6.155	3.45	7.533	4.21	*0.513*	7.046	4.02	6.26	5.14	*0.482*
LFH	67.42	6.21	67.13	4.91	*0.396*	58.06	5.87	59.52	4.82	*0.271*	69.11	9.24	68	8.80	*0.372*
CO-GN	110.3	4.79	115.8	4.89	***0.001***	96.72	8.72	101.6	7.28	***0.001***	118.9	12.6	118.4	13.93	***0.004***
1+SN	105.5	9.28	103.4	5.11	***0.031***	110	7.67	107.3	6.36	***0.003***	107	4.78	105.9	5.78	***0.001***
IMPA	95.07	6.67	94.74	6.89	*0.863*	94.79	3.95	100.9	3.42	***0.004***	90.85	6.43	91.18	7.18	*0.354*
Overbite	1.31	1.67	2.97	1.35	***0.001***	3.66	1.98	2.850	1.75	***0.006***	3.37	3.16	3.72	1.40	***0.002***
Overjet	6.89	1.96	4.34	1.24	***0.001***	7.66	1.90	4.09	1.05	***0.02***	6.31	2.55	2.7	2.51	***0.005***
ANB	5.61	1.43	2.39	0.85	***0.002***	6.22	1.72	4.51	1.47	**0.001**	7.61	2.50	6.60	2.09	***0.05***

**Table 4:** One-way ANOVA test for all the cephalometric variables between the three groups.

		Sum of Squares	df	Mean Square	F	Sig.
SN-PO	Between groups	612.614	2	306.307	21.694	0.001 *
	Within groups	1228.396	87	14.119		
	Total	1841.010	89			
SN-MP	Between groups	49.653	2	24.827	3.940	0.023 *
	Within groups	548.180	87	6.301		
	Total	597.833	89			
AR-GO-ME	Between groups	85.784	2	42.892	1.521	0.224
	Within groups	2453.685	87	28.203		
	Total	2539.469	89			
AR-GO-N	Between groups	28.064	2	14.032	1.143	0.323
	Within groups	1067.697	87	12.272		
	Total	1095.761	89			
N-GO-ME	Between groups	9.438	2	4.719	0.414	0.662
	Within groups	990.522	87	11.385		
	Total	999.960	89			
SN-PP	Between groups	57.155	2	28.578	4.273	0.017 *
	Within groups	581.825	87	6.688		
	Total	638.980	89			
LOWER FACE HEIGHT	Between groups	487.326	2	243.663	18.481	0.001 *
	Within groups	1147.030	87	13.184		
	Total	1634.355	89			
CO-GN	Between groups	344.719	2	172.359	4.429	0.015 *
	Within groups	3386.049	87	38.920		
	Total	3730.767	89			
1+SN	Between groups	257.450	2	128.725	3.431	0.037 *
	Within groups	3264.500	87	37.523		
	Total	3521.950	89			
IMPA	Between groups	743.330	2	371.665	21.883	0.001 *
	Within groups	1477.603	87	16.984		
	Total	2220.934	89			
OVERBITE	Between groups	71.946	2	35.973	6.850	0.002 *
	Within groups	456.915	87	5.252		
	Total	528.861	89			
OVERJET	Between groups	377.546	2	188.773	39.320	0.001 *
	Within groups	417.678	87	4.801		
	Total	795.224	89			
ANB	Between groups	72.458	2	36.229	21.511	0.001 *
	Within groups	146.526	87	1.684		
	Total	218.984	89			

* *p* < 0.05.

**Table 5:** Tukey’s post hoc test.

Dependent Variable	(I) GROUP	(J) GROUP	Mean Difference (I − J)	Std Error	Sig.	95% Confidence Interval	
						Lower Bound	Upper Bound
SN-PO	C	R	−2.77 *	0.970	0.015	−5.088	−0.461
	C	H	−6.37 *	0.970	0.000	−8.686	−4.059
	R	H	−3.59 *	0.970	0.001	−5.911	−1.284
SN-MP	C	R	1.81 *	0.648	0.017	0.269	3.360
	C	H	0.79	0.648	0.438	−0.747	2.343
	R	H	−1.01	0.648	0.264	−2.562	0.528
AR-GO-ME	C	R	−2.22	1.371	0.243	−5.490	1.050
	C	H	−1.88	1.371	0.360	−5.150	1.390
	R	H	0.34	1.371	0.967	−2.930	3.610
AR-GO-N	C	R	−1.12	0.904	0.434	−3.277	1.037
	C	H	0.12	0.904	0.990	−2.037	2.277
	R	H	1.24	0.904	0.361	−0.917	3.397
N-GO-ME	C	R	−0.55	0.871	0.803	−2.627	1.527
	C	H	−0.77	0.871	0.652	−2.847	1.307
	R	H	−0.22	0.871	0.965	−2.297	1.857
SN-PP	C	R	−0.92	0.667	0.356	−2.513	0.671
	C	H	−1.95 *	0.667	0.012	−3.543	−0.358
	R	H	−1.03	0.667	0.276	−2.622	0.562
LOWER FACE HEIGHT	C	R	5.46 *	0.937	0.000	3.231	7.702
	C	H	1.33	0.937	0.332	−0.898	3.572
	R	H	−4.13 *	0.937	0.000	−6.365	−1.894
CO-GN	C	R	−2.33 *	1.610	0.023	0.494	8.175
	C	H	0.39	1.610	0.967	−3.445	4.235
	R	H	−3.94 *	1.610	0.043	−7.780	−0.099
1+SN	C	R	3.25	1.581	0.105	−0.521	7.021
	C	H	3.85 *	1.581	0.044	0.078	7.621
	R	H	0.60	1.581	0.924	−3.171	4.371
IMPA	C	R	0.66	1.064	0.809	−1.876	3.198
	C	H	−5.73 *	1.064	0.000	−8.276	−3.201
	R	H	−6.40 *	1.064	0.000	−8.937	−3.862
OVERBITE	C	R	1.11	0.591	0.152	−0.301	2.521
	C	H	2.19 *	0.591	0.001	0.779	3.601
	R	H	1.08	0.591	0.167	−0.331	2.491
OVERJET	C	R	3.59 *	0.565	0.000	2.241	4.939
	C	H	4.83 *	0.565	0.000	3.481	6.179
	R	H	1.24	0.565	0.078	−0.109	2.589
ANB	C	R	2.15 *	0.335	0.000	1.351	2.949
	C	H	0.68	0.335	0.111	−0.119	1.479
	R	H	−1.47 *	0.335	0.000	−2.269	−0.671

* *p* < 0.05.

## Data Availability

The data presented in this study are available upon request from the corresponding author.
